# Guillain-Barré Syndrome as the Initial Manifestation of Lyme Disease: Diagnostic Challenges

**DOI:** 10.7759/cureus.105552

**Published:** 2026-03-20

**Authors:** Ahmed Elnour, Naveed Sultan, Abdul Monem, Khalid Ghalib

**Affiliations:** 1 Internal Medicine, University Hospital Kerry, Tralee, IRL

**Keywords:** bilateral facial palsy, borrelia burgdorferi, guillain-barré syndrome, lyme disease, lyme neuroborreliosis

## Abstract

Lyme disease is a common tick-borne infection in the United States and Europe that may involve the nervous system during the disseminated stage. Guillain-Barré syndrome (GBS) is an acute immune-mediated polyneuropathy usually triggered by infection; however, its association with *Borrelia burgdorferi* is uncommon and can pose diagnostic challenges.

We report the case of a previously healthy 61-year-old female patient who presented with progressive ascending weakness and areflexia suggestive of GBS. During hospitalisation, she developed bilateral facial nerve palsy, prompting further evaluation. Cerebrospinal fluid (CSF) findings and electrophysiological studies supported acute inflammatory demyelinating polyneuropathy, while serologic testing confirmed Lyme disease. The patient received intravenous immunoglobulin (IVIG) followed by intravenous ceftriaxone and achieved complete neurological recovery.

This case emphasises the need to consider Lyme disease in patients presenting with acute inflammatory neuropathy, particularly in endemic regions, as early diagnosis and targeted therapy can significantly improve outcomes.

## Introduction

Guillain-Barré syndrome (GBS) is an acute immune-mediated polyneuropathy typically characterised by progressive, ascending weakness [1]. It is typically preceded by an infectious or immunological trigger. Among the infectious triggers most frequently implicated are *Campylobacter jejuni*, cytomegalovirus, Epstein-Barr virus, human immunodeficiency virus, and Zika virus infection [2, 3]. It is typically preceded by an infectious or immunological trigger that provokes an aberrant immune response directed against peripheral nerves [1, 4]. However, GBS associated with tick-borne pathogens, including *Borrelia burgdorferi*, has been reported only in rare cases [1, 2, 5]. Lyme disease is a common tick-borne spirochaetal infection caused by one of several *Borrelia *species, most commonly *Borrelia burgdorferi *and *Borrelia garinii* [2, 4-8]. It is a multisystem illness that typically progresses through three clinical stages: early localised, early disseminated, and late disease [5, 7-9]. Neurological involvement occurs during the disseminated phase and may manifest as Lyme neuroborreliosis. The most common neurological manifestations include cranial neuropathies (particularly facial nerve palsy), lymphocytic meningitis, and radiculoneuritis [4]. Approximately 40% of affected individuals develop neurological complications during the course of the disease [2].

## Case presentation

A 61-year-old previously healthy woman presented to the accident and emergency department with a two-week history of generalised fatigue. One day before the presentation, she developed acute bilateral lower limb weakness associated with distal paraesthesia. She denied facial or upper limb weakness at onset and reported no dysarthria, visual disturbance, or respiratory or gastrointestinal symptoms. There was no recent travel history or reported insect bites.

On initial examination, she was conscious and oriented to time, place, and person. Her vitals were unremarkable. Motor assessment revealed bilateral symmetrical weakness in the lower limbs, graded 3/5, with distal involvement more pronounced than proximal weakness (proximal power 4/5). Ankle and knee reflexes were absent bilaterally. Tone was reduced bilaterally, while sensory examination remained intact. Cranial nerve and upper limb examinations were unremarkable.

**Figure 1:**
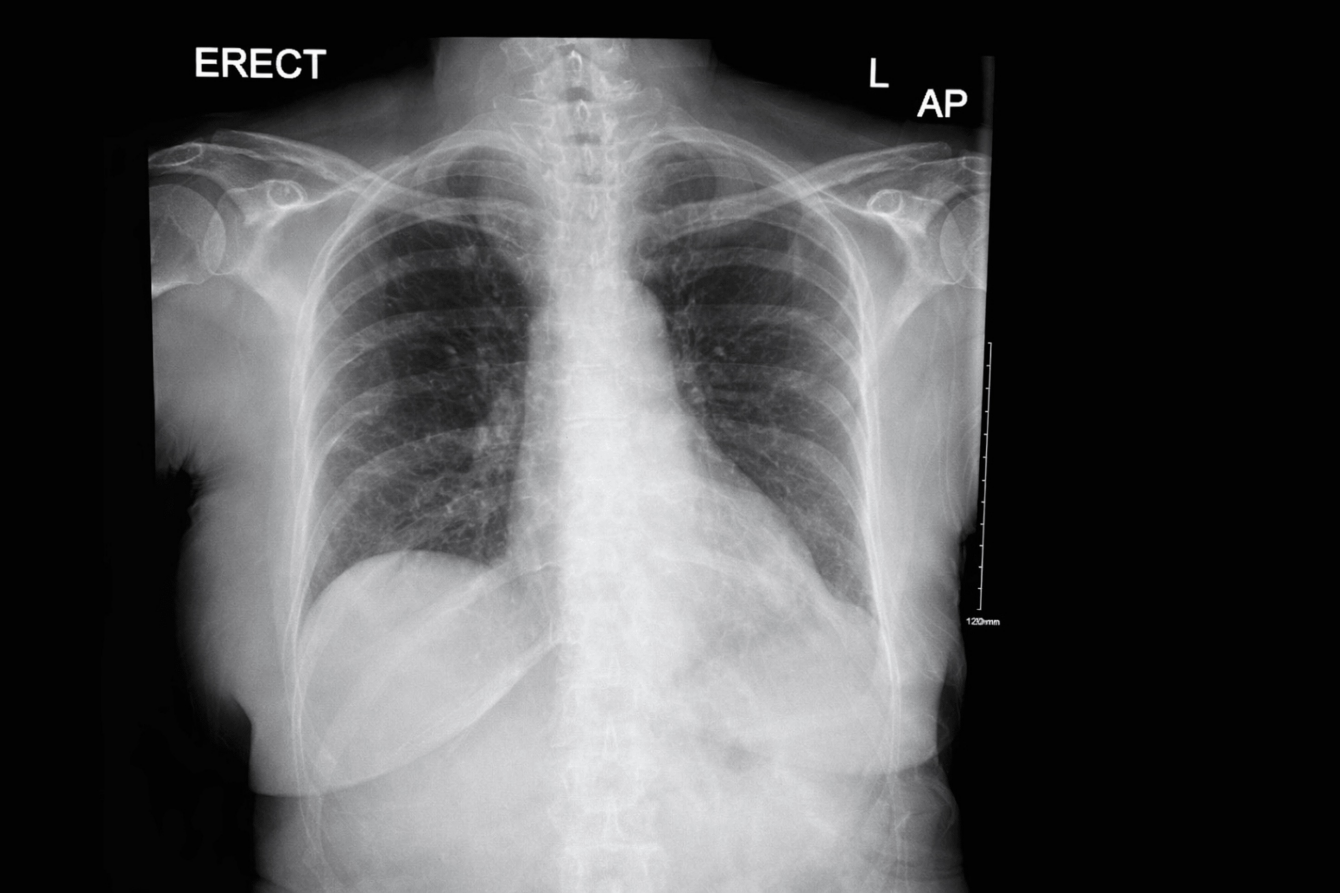
The Patient's Chest Radiograph Normal Cardiac and Mediastinal Contours With Clear Lung Fields. No Focal Consolidation, Pleural Effusion, Pneumothorax, or Acute Cardiopulmonary Abnormality Was Identified.

Initial laboratory investigations, including a complete blood count (Table 1) and basic metabolic panel (Table 2), were largely unremarkable, except for mild leukocytosis and an elevated C-reactive protein level, suggesting a possible inflammatory or infectious trigger. Chest radiography revealed no acute cardiopulmonary pathology (Figure 1).

**Table 1:** Complete Blood Count (CBC) WBC: White Blood Cell Count; RBC: Red Blood Cell Count; Hb: Haemoglobin; HCT: Haematocrit; MCV: Mean Corpuscular Volume; MCH: Mean Corpuscular Haemoglobin; MCHC: Mean Corpuscular Haemoglobin Concentration; RDW: Red Cell Distribution Width

Test	Result	Units	Reference Range
WBC	12.6 ↑	x10⁹/L	4.4–11.3
RBC	5.11	x10¹²/L	3.9–5.3
Hb	15.1	g/dL	11.7–15.9
HCT	0.445	L/L	0.35–0.46
MCV	87.1	fL	80–96
MCH	29.5	pg	26–34
MCHC	33.9	g/dL	31–37
RDW	12.1	%	11.6–15
Platelets	292	x10⁹/L	140–440
Neutrophils	2.74	x10⁹/L	1.4–6.6
Lymphocytes	8.56 ↑	x10⁹/L	0.9–3.2
Monocytes	1.05	x10⁹/L	0.15–1.3
Eosinophils	0.06	x10⁹/L	0.04–0.4
Basophils	0.18	x10⁹/L	0–0.1

**Table 2:** Basic Metabolic Panel AST: Aspartate Aminotransferase; ALT: Alanine Aminotransferase; GGT: Gamma-Glutamyl Transferase; TSH: Thyroid-Stimulating Hormone; T4: Thyroxine

Test	Result	Units	Reference Range
Sodium	137	mmol/L	133–146
Potassium	4.3	mmol/L	3.5–5.3
Chloride	99	mmol/L	95–108
Urea	3.6	mmol/L	2.5–7.8
Creatinine	56	µmol/L	49–90
Total Protein	82 ↑	g/L	60–80
Albumin	37	g/L	35–50
AST	39 ↑	U/L	1–35
ALT	35	U/L	1–35
Alkaline Phosphatase	116	U/L	40–120
GGT	37	U/L	1–38
Total Bilirubin	13	µmol/L	5–21
Calcium	2.35	mmol/L	2.20–2.65
Phosphate	0.98	mmol/L	0.80–1.50
Magnesium	0.92	mmol/L	0.70–1.00
C-Reactive Protein	20.2 ↑	mg/L	0.2–5.0
Free T4	13.7	pmol/L	8–16
TSH	0.61	mIU/L	0.38–5.33

Lumbar puncture and cerebrospinal fluid (CSF) analysis (Table 3) demonstrated albuminocytologic dissociation-elevated CSF protein with a normal white blood cell count-a characteristic finding supporting the diagnosis of GBS.

**Table 3:** CSF Analysis CSF: Cerebrospinal Fluid; WCC: White Cell Count

Parameter	Result	Units	Reference Range
CSF WCC	1	/µL	0–6 /µL
CSF Glucose	3.9	mmol/L	Greater than 60% of the serum glucose
Serum Glucose	6.8	mmol/L	3.9–7.8 mmol/L
CSF Protein	51.6 ↑	mg/dL	15–45 mg/dL
Extended CSF Viral Panel	Negative		Negative

**Figure 2:**
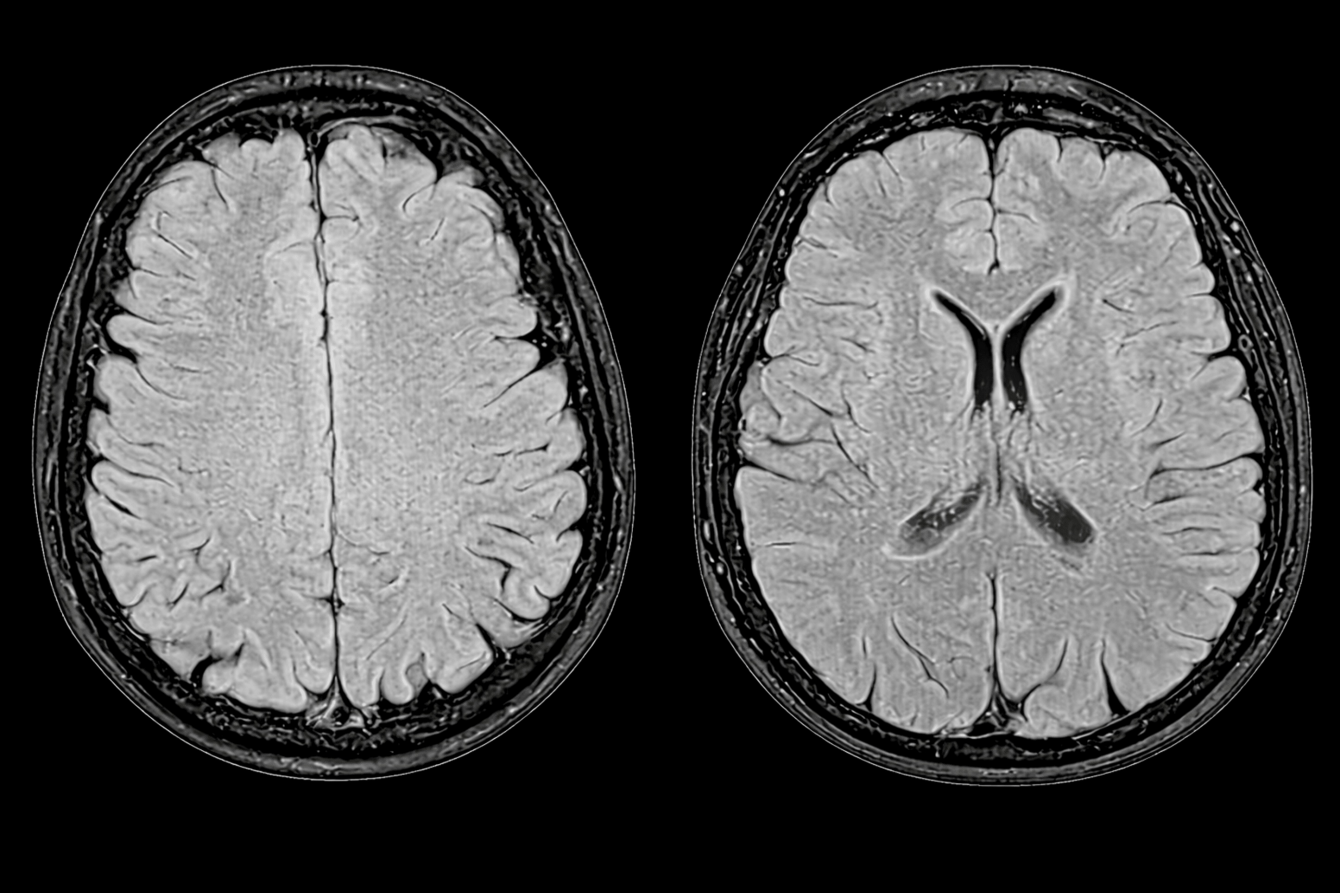
Magnetic Resonance Imaging (MRI) of the Brain Normal Brain Parenchyma and Ventricular System; No Focal Structural Abnormality Was Noted.

Magnetic resonance imaging of the brain was normal (Figure 2). Cervical and lumbosacral spine imaging were unremarkable.

A ganglioside antibody panel was performed and demonstrated the presence of anti-ganglioside antibodies, including a positive anti-GD2 IgM antibody with a titre graded as (++), as shown in Table 4. This result was interpreted alongside the clinical and neurophysiological findings.

**Table 4:** Ganglioside Antibody Panel AMAN: Acute Motor Axonal Neuropathy; Anti-GD2 Antibodies: Antibodies Directed Against GD2 Ganglioside; IgM: Immunoglobulin M; NCS: Nerve Conduction Studies

Parameters	Patient Result	Normal Reference	Clinical Interpretation
Anti-ganglioside Antibodies	Present	Negative	Indicates the Presence of at Least One Ganglioside Antibody; Not Diagnostic Alone
Anti-GD2 Antibody	Positive	Negative	Not a Classic GBS Antibody; Low Titre (+) Often Non-specific. Higher Titres (++/+++) Are More Significant.
Isotype	IgM	No Detectable IgM Anti-ganglioside Antibodies	IgM is More Often Seen in AMAN and Some Immune Neuropathies; Requires Clinical/NCS Correlation
Titre Grading	(++)	Negative	(+) = Weak/Low Significance; (++/+++) Stronger Pathological Association

Nerve conduction studies and electromyography were performed. Sensory nerve conduction studies demonstrated normal sensory nerve action potentials in the right median, radial, ulnar, superficial peroneal, and sural nerves.

Motor nerve conduction studies showed normal compound muscle action potentials in the right median nerve recorded from the abductor pollicis brevis muscle, the right ulnar nerve recorded from the abductor digiti minimi muscle, the right peroneal nerve recorded from the extensor digitorum brevis muscle, and the right tibial nerve recorded from the abductor hallucis muscle.

Assessment of late responses demonstrated absent F-waves in the right tibial nerve, while right ulnar F-waves were preserved.

The absence of F-wave responses on nerve conduction studies is consistent with early acute demyelinating neuropathy and provides electrophysiological support for acute inflammatory demyelinating polyneuropathy.

Concentric needle electromyography of selected muscles from the right upper and lower limbs, as well as facially innervated muscles, revealed no abnormal spontaneous activity. Occasional fast-firing motor unit potentials were observed in the right triceps, first dorsal interosseous muscle, and facial muscles, including orbicularis oculi and orbicularis oris.

Overall, the neurophysiological findings were abnormal and suggestive of a mild acquired demyelinating neuropathy with early electrophysiological recovery.

A diagnosis of acute inflammatory demyelinating polyneuropathy was considered, and a five-day course of intravenous immunoglobulin (IVIG) was administered. On hospital day three, she developed progressive ascending weakness involving the upper limbs and bilateral lower motor neuron facial palsy. Lyme disease was suspected, and serologic testing returned positive (Table 5), fulfilling the Centers for Disease Control and Prevention (CDC) two-tier diagnostic criteria and indicating exposure to* Borrelia burgdorferi* in the appropriate clinical context [10].

**Table 5:** Lyme Disease Serology IgG: Immunoglobulin G; IgM: Immunoglobulin M; B. burgdorferi IgG/IgM (EIA): *Borrelia burgdorferi* Immunoglobulin G and Immunoglobulin M Detected by Enzyme Immunoassay. According to the Centers for Disease Control and Prevention (CDC) Two-Tier Testing Criteria [10], Serologic Confirmation of Lyme Disease Requires a Positive or Equivocal Enzyme Immunoassay (EIA) Followed by a Positive Immunoblot. In the Present Case, Both IgM and IgG Line Blot Assays Returned Positive Results, Fulfilling CDC Criteria for Serologic Confirmation of *Borrelia burgdorferi* Exposure.

Test	Result	Reference
*Borrelia *IgM Line Blot	Positive	Negative
*Borrelia *IgG Line Blot	Positive	Negative
*Borrelia burgdorferi* IgG	Positive	Negative
*Borrelia burgdorferi *IgG/IgM (EIA)	Positive	Negative

Consequently, oral doxycycline was initiated but discontinued due to gastrointestinal intolerance. The patient was subsequently switched to intravenous ceftriaxone for a 30-day course. Two weeks after completing intravenous therapy, she achieved complete clinical recovery and returned to her baseline level of functional independence.

Respiratory function was closely monitored. Peak flow rate was initially 320 L/min (reference: 400-500 L/min), then improved to 470 L/min at discharge. Forced vital capacity was 2300 mL (50 mL/kg; reference: 60-70 mL/kg) on admission and improved to 3900 mL (62 mL/kg) at discharge. Negative inspiratory force measured -42 cmH₂O (reference: -60 to -100 cmH₂O), which improved to -63 at discharge. The patient did not require intubation or ventilatory support.

Following five days of IVIG therapy combined with intensive physiotherapy and intravenous ceftriaxone, the patient exhibited noticeable neurological improvement. She regained partial ambulatory ability and was able to mobilise with a Zimmer frame with the assistance of a single person. Given her continued recovery, she was discharged to a rehabilitation facility for further physiotherapy and functional rehabilitation.

## Discussion

GBS has an estimated global annual incidence of approximately one in 100,000 cases [1, 7]. It is typically triggered by an infection or immune response that attacks the peripheral nerves. Although *Campylobacter jejuni*, cytomegalovirus, and Epstein-Barr virus are common causes, the connection with *Borrelia burgdorferi *is rare [1, 11]. Lyme disease can impact the nervous system during its disseminated phase, resulting in symptoms such as cranial neuropathies, radiculopathy, or peripheral neuropathy [2, 5, 7].

In this case, the patient initially demonstrated classical features of acute inflammatory demyelinating polyneuropathy, including ascending symmetrical weakness, areflexia, and albuminocytologic dissociation. Respiratory involvement further supported the diagnosis of GBS [12, 13]. The patient showed reduced peak flow, forced vital capacity, and negative inspiratory force, consistent with early neuromuscular respiratory compromise; however, she did not require mechanical ventilation [12, 13].

The subsequent development of bilateral lower motor neuron facial palsy broadened the differential diagnosis, as, although rare overall, it is a recognised manifestation of Lyme neuroborreliosis and therefore appropriately prompted serologic evaluation [8,9,11].

It is believed that following *Campylobacter* infection, the host immune response generates autoantibodies directed against ganglioside-like lipooligosaccharides expressed on the bacterial surface [1, 13].

Similarly, there is a possible association between *Borrelia burgdorferi *and GBS, with the bacterium acting as an antigen or immune complex that induces the production of anti-ganglioside antibodies, leading to GBS in susceptible individuals [1, 4]. Although anti-GD2 antibodies are not among the classical antibodies associated with GBS, their presence may reflect immune activation and has been described in some immune-mediated neuropathies [4, 13]. In this case, the finding was interpreted in conjunction with the clinical presentation and electrophysiological evidence supporting acute inflammatory demyelinating polyneuropathy.

Serology fulfilled the CDC, with positive enzyme immunoassay and confirmatory IgM and IgG line blot [10, 14]. Interpretation of Lyme serology should always be made in the clinical context, as IgG positivity alone may reflect previous exposure in endemic areas rather than active infection [15]. In this case, both IgM and IgG antibodies were detected during the period of acute neurological deterioration. The onset of bilateral lower motor neuron facial palsy prompted serologic testing and raised suspicion for Lyme neuroborreliosis [1, 9, 11]. Taken together with the patient’s subsequent clinical improvement after treatment with intravenous ceftriaxone, these findings support a likely temporal relationship between *Borrelia *infection and the neurological syndrome.

The diagnosis of GBS was further supported by nerve conduction studies, which demonstrated absent F-wave responses, consistent with an acute demyelinating neuropathy [9, 13].

The patient showed partial improvement with the early initiation of IVIG. A complete recovery was achieved after she received intravenous ceftriaxone for one month. The patient was unable to tolerate oral doxycycline; consequently, therapy was switched to intravenous ceftriaxone as an alternative, which was better tolerated. Shrestha et al. reported the case of a 65-year-old gentleman with GBS secondary to Lyme disease, who only improved after receiving intravenous ceftriaxone [7, 16].

This was supported by a multicentre, randomised, open-label equivalence trial that demonstrated that oral doxycycline is as effective as intravenous ceftriaxone in the treatment of Lyme neuroborreliosis [17]. Both treatment groups showed comparable clinical improvement at 12 months, with no significant differences in subjective recovery or CSF parameters [17]. These findings support the use of oral doxycycline as a safe, effective, and practical alternative to intravenous therapy, offering advantages in cost, convenience, and avoidance of intravenous-related complications [5, 17].

## Conclusions

This case highlights the diagnostic complexity of distinguishing GBS from Lyme neuroborreliosis when clinical features overlap. Although GBS and Lyme neuroborreliosis may share overlapping neurological signs, the presence of progressive ascending weakness, generalised areflexia, albuminocytologic dissociation, and supportive electrophysiological findings in this case favoured a diagnosis of GBS in association with *Borrelia burgdorferi* infection. The presence of bilateral facial palsy in the context of acute demyelinating polyneuropathy appropriately prompted testing for *Borrelia burgdorferi*, ultimately supporting a diagnosis of GBS occurring in association with Lyme disease. Early immunomodulatory therapy with IVIG and targeted antimicrobial treatment resulted in complete neurological recovery.

Clinicians should maintain a high index of suspicion for Lyme disease in patients presenting with atypical GBS features, particularly in endemic regions, as timely recognition and combined therapeutic strategies can significantly improve outcomes.
